# Pathogenic genotype of major piroplasm surface protein associated with anemia in *Theileria orientalis* infection in cattle

**DOI:** 10.1186/s13028-017-0318-8

**Published:** 2017-07-27

**Authors:** Suhee Kim, Do-Hyeon Yu, Jeong-Byoung Chae, Kyoung-Seong Choi, Hyeon-Cheol Kim, Bae-Keun Park, Joon-Seok Chae, Jinho Park

**Affiliations:** 1 National Institute of Animal Science, Rural Development Administration, Wanju, 55365 Republic of Korea; 2 College of Veterinary Medicine, Gyeongsang National University, Jinju, 52828 Republic of Korea; 3 Laboratory of Veterinary Internal Medicine, BK21 PLUS Program for Creative Veterinary Science Research, Research Institute for Veterinary Science and College of Veterinary Medicine, Seoul National University, Seoul, 08826 Republic of Korea; 4 College of Ecology and Environmental Science, Kyungpook National University, Sangju, 37224 Republic of Korea; 5 College of Veterinary Medicine, Kangwon National University, Chuncheon, 24341 Republic of Korea; 6 College of Veterinary Medicine, Chungnam National University, Daejeon, 34134 Republic of Korea; 7 College of Veterinary Medicine, Chonbuk National University, Iksan, 54596 Republic of Korea

**Keywords:** Anemia, Major piroplasm surface protein, MPSP type 1, *Theileria orientalis*

## Abstract

Serious disease outbreaks in cattle caused by *Theileria orientalis* have emerged in the Asia–Pacific region. Genetic variables of the major piroplasm surface protein (MPSP) expressed on the surface of the piroplasm inside *T. orientalis*-infected erythrocytes are considered to be associated with variation in the pathogenicity of *T. orientalis*. Our study describes the clinically relevant MPSP types associated with anemia in *Theileria*-infected cattle. These results revealed that MPSP expression plays an important role in hematological alterations in *Theileria*-infected cattle, and that MPSP type 1 is strongly associated with bovine anemia, which can be a potential target for the prevention of bovine theileriosis.

## Findings

Bovine theileriosis, caused by subspecies of the protozoan parasite *Theileria*, is one of the most economically important diseases of cattle throughout various regions of the world [1]. *Theileria parva* and *T. annulata*, commonly known as East Coast fever and tropical theileriosis, respectively, are highly pathogenic, whereas *T. orientalis* is believed to cause mild or asymptomatic disease [2]. However, recently, *T. orientalis* has emerged as an agent capable of causing outbreaks of clinical theileriosis resulting in losses to the Asia–Pacific cattle industry [3, 4].

*Theileria orientalis* proliferates inside erythrocytes as piroplasms [5]. During the intraerythrocytic stage, *T. orientalis* can cause hemolysis and subsequent anemia, which is the primary clinical finding [6, 7]. The major piroplasm surface protein (MPSP), conserved to the piroplasms of *T. orientalis* is expressed in the intraerythrocytic stage of the parasite [8]. The sequence variations in the MPSP gene have been used to define the genetic diversity of *T. orientalis* [9]. Currently, 11 genotypes of *T. orientalis* (types 1–8 and N1–N3) have been identified based on the MPSP gene sequence [9].

Despite few studies on pathogenicity of MPSP types [10], most studies are still focused on epidemiological investigations that identify and characterize the genotypes of *T. orientalis* [8, 9, 11, 12]. There is limited information on disease outbreaks related to the genotypes of *T. orientalis* [3, 10, 13] and the clinical relevance of the various MPSP types has not been clearly elucidated.

In a recent study, various MPSP types (types 1, 2, 3, and 7) were identified in the Republic of Korea [14]. Based on the MPSP genotypes identified from our previous report [14], we investigated the genotypes of *T. orientalis* associated with clinical disease by evaluating changes in red blood cell (RBC) profiles according to different MPSP types in *Theileria*-infected cattle. The objective of this study was to determine the main *T. orientalis* MPSP genotypes associated to disease severity and development of anemia in cattle.

A total of 143 Holstein cattle from three geographical regions of the Republic of Korea (Hoengseong, Namwon, and Jeju island) were randomly selected between April and September 2015. Blood samples for hematological analysis and *Theileria* polymerase chain reaction (PCR) analysis were collected from the jugular vein into ethylenediaminetetraacetic acid (EDTA) tubes. A RBC profile, including RBC count, hemoglobin (Hb) level, hematocrit (HCT), mean cell volume (MCV), mean corpuscular hemoglobin (MCH), and mean corpuscular hemoglobin concentration (MCHC) was created using an automatic blood analyzer (Hemavet 960, Erba Diagnostics Inc., Miami, FL, USA). Cattle were divided into three categories based on RBC, Hb, and HCT values according to [8]. The categories were: severely anemic (RBC <3.0 M/μL, Hb <5.0 g/dL or HCT <15%); mildly anemic (RBC 3.0–5.0 M/μL, Hb 5.0–8.0 g/dL or HCT 15–24%), and without anemia (RBC >5.0 M/μL, Hb >8.0 g/dL or HCT >24%).

The *T. orientalis* types obtained have been reported previously [14]. Briefly, to identify *T. orientalis* infection, genomic DNA was extracted from whole blood using the DNeasy Blood & Tissue Kit (Qiagen Inc., Valencia, CA, USA). PCR was performed on all samples using a primer set targeting the 18S ribosomal RNA (rRNA) gene of *Theileria* [15]. In samples positive for the *Theileria*-18S rRNA gene, PCR was performed on the MPSP gene of *T. orientalis* [14]. To identify *T. orientalis* genotypes, DNA sequencing and phylogenetic analysis were performed on samples positive for the MPSP gene [14]. A total of four MPSP types (types 1, 2, 3 and 7) were identified, and deposited accession numbers were described in previous publication [14].

We used a SPSS 23.0 software package (SPSS, Chicago, IL, USA) to analyze data. The Shapiro–Wilk test was utilized for normality analysis. To compare alterations in the RBC profile according to 18S or MPSP gene expression, either an independent *t* test or Mann–Whitney *U* test was performed, depending on the results of a normality test. To compare anemic parameters among different MPSP genotypes, one-way analysis of variance (ANOVA) or Kruskal–Wallis was used accompanied by least significant difference (LSD) or Mann–Whitney test depending on the normal distribution of data. Data are expressed as mean ± standard deviation (SD) and a P value of <0.05 was considered significant.

To determine whether antigenic expression in erythrocytes infected with *T. orientalis* was a critical factor for progression of clinical disease, RBC profiles were compared among cattle positive for *Theileria*-18S or the *T. orientalis*-MPSP gene to cattle negative for *Theileria*-18S (non-infected with *Theileria*) or *T. orientalis*-MPSP gene (infected with *Theileria* but without MPSP expression) (Table 1). Cattle positive for the *Theileria*-18S gene had decreased RBC and HCT and increased MCV, MCH, and MCHC when compared to cattle negative for the *Theileria*-18S gene (P < 0.05). Of cattle positive for the *Theileria*-18S gene, RBC, Hb, and HCT of MPSP-positive cattle were significantly lower than those of MPSP-negative cattle (P < 0.005) or non-infected cattle (P < 0.01). In addition, the MCV and MCH of MPSP-positive cattle were significantly higher than found in MPSP-negative cattle (P < 0.005) or non-infected cattle (P < 0.05). In contrast, there were no differences in the RBC profile of MPSP-negative cattle when compared to non-infected cattle. Hematological alterations in *Theileria*-18S-positive cattle were dependent on the expression of the MPSP gene. This result revealed that detection of MPSP gene is a useful biomarker to predict development of clinical disease in *Theileria*-infected cattle.

**Table 1:** Alterations in the red blood cell profile of cattle according to antigenicity of *Theileria orientalis*

RBC profile	n	RBC (M/µL)	Hb (g/dL)	HCT (%)	MCV (fL)	MCH (pg)	MCHC (g/dL)
Reference values		5.0–10.0	8.0–15.0	24.0–46.0	40.0–60.0	11.1–17.0	28.2–36.0
18S negative	53	9.6 ± 1.6	10.0 ± 1.5	32.4 ± 5.5	34.4 ± 3.9	10.6 ± 1.8	31.1 ± 4.6
18S positive	90	8.2 ± 2.1**	9.9 ± 3.3	30.2 ± 6.5*	37.6 ± 5.7**	12.3 ± 2.6**	32.5 ± 4.7*
MPSP negative	34	9.8 ± 0.8	10.6 ± 0.5	32.5 ± 2.0	33.1 ± 2.1	10.5 ± 0.7	31.8 ± 1.5
MPSP positive	56	7.3 ± 2.1**^a^	9.6 ± 4.1**^a^	28.9 ± 7.8**^a^	39.5 ± 5.7**^a^	13.1 ± 2.7**^a^	32.8 ± 5.5*

* P < 0.05 and ** P < 0.01 vs. 18S negative, ^a^ P < 0.005 vs. major piroplasm surface protein (MPSP) negative

**Fig. 1:**
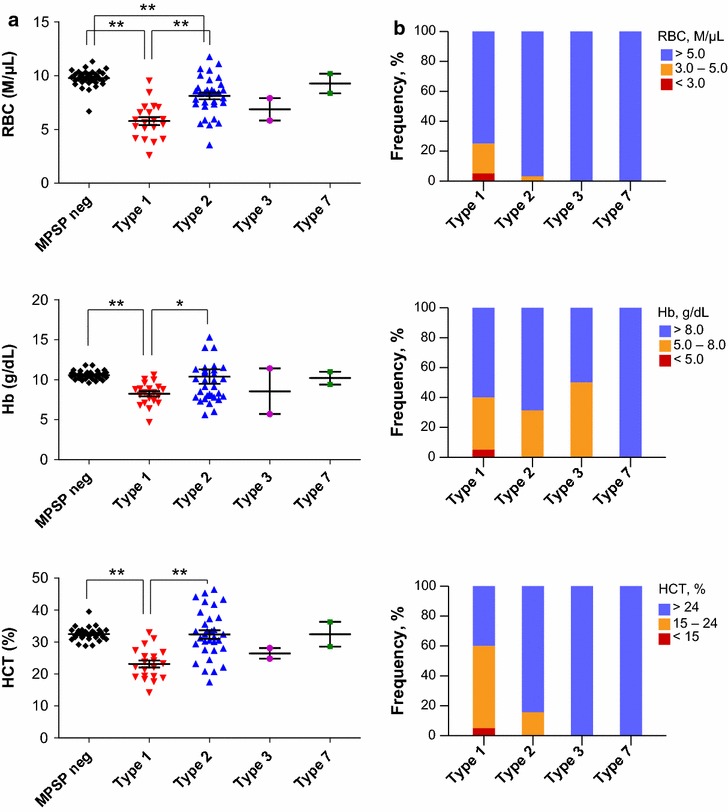
Development of anemia according to major piroplasm surface protein (MPSP) genotypes in *Theileria*-infected cattle. Comparison of red blood cell count (RBC), hemoglobin (Hb), and hematocrit (HCT) values among MPSP types (**a**) and the presence of anemia in each MPSP type (**b**). *Colored bars* indicated the level of anemia. *Blue* no anemia; *orange* mild anemia and *red* severe anemia. Number of animals: MPSP-negative (n = 34); types 1 (n = 20), 2 (n = 32), 3 (n = 2) and 7 (n = 2). *P < 0.05 and **P < 0.001

To investigate clinically relevant MPSP types, MPSP type-specific hematological changes and development of anemia were investigated in *T. orientalis*-infected cattle (Fig. 1). Of the four MPSP types identified, the RBC, Hb, and HCT values of type 1-infected cattle were significantly lower than those of MPSP-negative cattle (P < 0.001) or type 2-infected cattle (P < 0.05) (Fig. 1a). Type 2-infected cattle showed a significant decrease in RBC (P < 0.001), but no difference in Hb or HCT, when compared to MPSP-negative cattle.

Type 1-infected cattle showed a greater proportion of severely and mildly anemic cattle when compared to cattle infected with other types (Fig. 1b). When the RBC count was used as the diagnostic criterium, severe or mild anemia was observed in 25% (n = 5) of the 20 type 1-infected cattle. However, only 3.1% (n = 1) of the 32 type 2-infected cattle had severe or mild anemia. Among type 1-infected cattle, 40% were anemic using the Hb criteria and 60% using the HCT criteria. Among type 2-infected cattle, 31.1 and 15.6% were anemic using the Hb and HCT criteria, respectively. There was a high incidence of anemia among type 1-infected cattle suggesting that infection with *T. orientalis* type 1 can be strongly associated with clinical disease.

The aim of this study was to explore if there was an association between the *T. orientalis* MPSP genotype and the development of anemia in *T. orientalis*-infected cattle. This is first report to show that *T. orientalis* type 1 is closely associated with anemia leading to clinical theileriosis. Although anemia associated with *T. orientalis* type 2-infection is less common than anemia associated with type 1-infection, this is also the first report of anemia associated with type 2-infection in the Republic of Korea.

This study has several limitations. First, due to the limited number of samples we were unable to determine and describe the pathogenicity of *T. orientalis* types 3 and 7 in detail. Based on other reports, types 3 and 5 are considered to be low pathogenic in cattle [3], while type 7 has been related to outbreaks of clinical disease in India [16]. A large-scale study with an increased number of samples is needed to explore this further. Second, pathogenic determinants for *T. orientalis* can include parasite load in addition to parasite genotype. High parasite loads correlate with clinical theileriosis. Type 2 infection with high parasite loads is negatively correlated with packed cell volume [6, 17]. Although we could not clearly elucidate whether clinical disease was a result of genotype, total parasite burden or a combination of genotype and burden, it was clear that type 1 infection was strongly linked to anemia in cattle. Further studies to determine critical factors responsible for clinical theileriosis in *T. orientalis* infection should include measurement of the parasite load in the blood.

Type 2 has been thought to be the key causal variant in bovine theileriosis [10]. Clinical cases of theileriosis were associated with type 2 alone or in combination with type 1 in Australia [10], while infection with type 1 alone was often associated with subclinical disease [17]. In contrast to previous reports, our study showed that type 1 infection alone can be a major causal factor for the outbreak of clinical theileriosis. Although several researches suggested that type 1 is a direct cause of disease [18, 19], the pathogenicity of type 1 is still less clear than that of type 2. Therefore, continuous monitoring of type 1-infected cattle is needed to assess the progression to clinical disease.

In conclusion, type 1 can be responsible for direct cause of disease outbreaks in cattle. Understanding the types of *T. orientalis* that result in theileriosis can aid in the development of effective strategies to prevent pathogenic orientalis theileriosis.

## Abbreviations

EDTA: ethylenediaminetetraacetic acid
Hb: hemoglobin
HCT: hematocrit
MCH: mean corpuscular hemoglobin
MCHC: mean corpuscular hemoglobin concentration
MCV: mean cell volume
MPSP: major piroplasm surface protein
PCR: polymerase chain reaction
RBC: red blood cell
rRNA: ribosomal RNA
*T. orientalis*: *Theileria orientalis*
